# Breast and splenic metastases of squamous cell carcinoma from the uterine cervix: a case report

**DOI:** 10.1186/1752-1947-8-359

**Published:** 2014-11-04

**Authors:** Meryem Aitelhaj, Siham L Khoyaali, Anouar Boukir, Mustapha Elkabous, Halima Abahssain, Hind Mrabti, Basma El Khannoussi, Hassan Errihani

**Affiliations:** 1 Medical Oncology Department, National Institute of Oncology, Quartier Irfane, Hay Riad 10080, Rabat, Morocco; 2 Pathology Department, National Institute of Oncology, Quartier Irfane, Hay Riad 10080, Rabat, Morocco

**Keywords:** Breast metastasis, Splenic metastasis, Cervical cancer

## Abstract

**Introduction:**

Metastases to the breast from extramammary malignancies are infrequent, the most common primary sites are malignant melanoma, leukemia, lymphoma, and cancer of the lung, stomach, prostate and ovary. The cervical origin is exceptional.

Splenic metastasis from squamous cell carcinoma of the cervix is also rare. To the best of our knowledge, only three cases of isolated splenic metastasis have been reported in the literature.

**Case presentation:**

We describe the case of a 55-year-old North African woman who presented with a nodule in her left breast eight months after treatment for stage IIB squamous cell uterine cervical carcinoma. The excisional biopsy with histological study demonstrated a poorly differentiated squamous cell carcinoma. A computed tomography scan revealed a splenic secondary location.

**Conclusions:**

We report here a case of two unusual metastatic sites of uterine cervical carcinoma, the breast and spleen. It is the first case of this association without widespread disease.

## Introduction

Primary breast carcinoma is the most common neoplasm in women. Whereas metastases to the breast from extramammary malignancies are extremely rare, a frequency of 0.5% to 6.6% has been reported in clinical and autopsy studies. The common primary sites in order of decreasing frequency are malignant melanoma, leukemia, lymphoma, and cancer of the lung, stomach, prostate and ovary [1,2]. The cervical origin is rarely reported, and often occurs in widespread disease with multiple other metastatic sites, notably lung.

Splenic metastasis from squamous cell carcinoma of the cervix is exceptional; to the best of our knowledge, only three cases of isolated splenic metastasis have been reported in the literature [3-5]. Here, we present the first case of the association of breast and splenic metastases from squamous cell carcinoma of the uterine cervix.

## Case presentation

**Figure 1:**
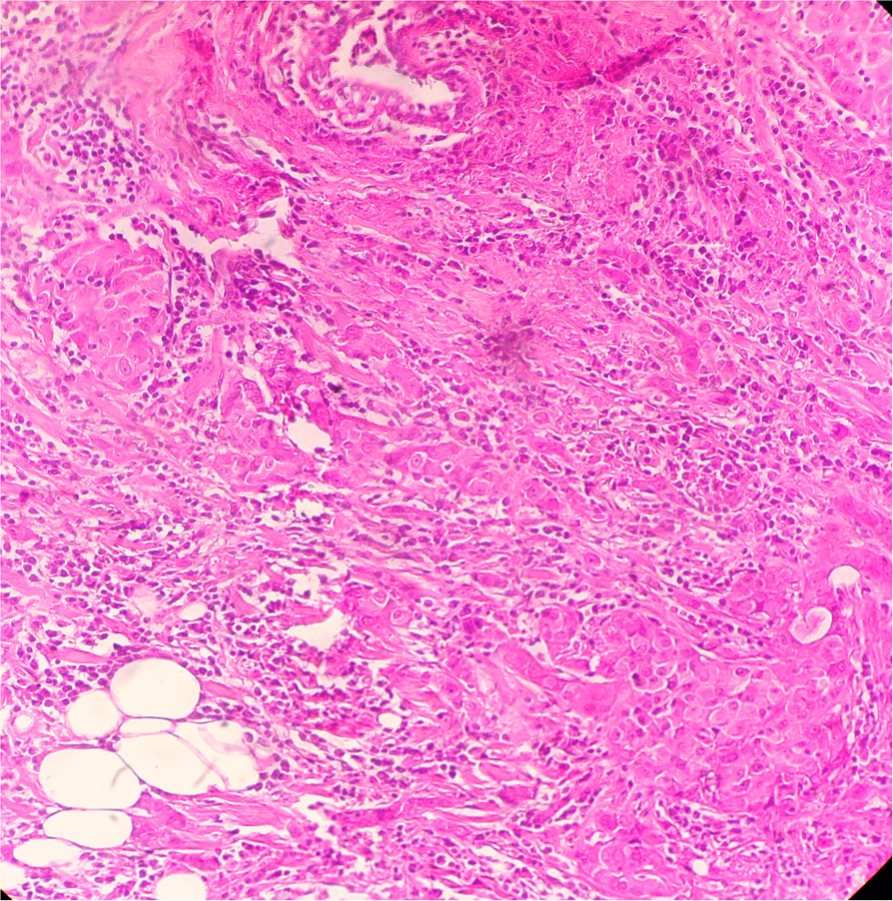
**Microphotography showing neoplastic cell proliferation organized in nests of tumor cells, within the mammary tissue.** Stain: hematoxylin and eosin; magnification: 100×.

**Figure 2:**
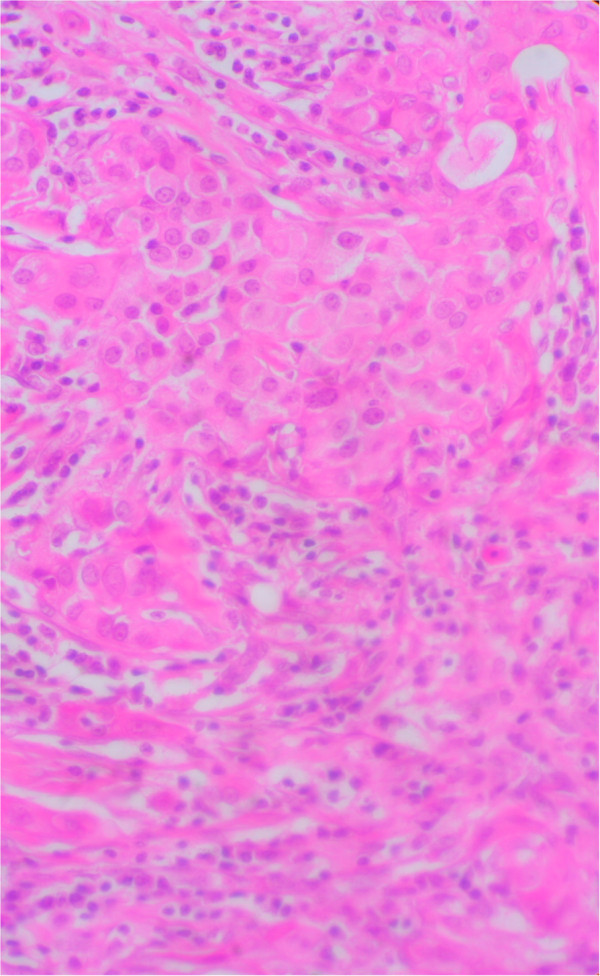
**Microphotography showing neoplastic cell proliferation organized in nests of tumor cells with rare union bridges.** Stain: hematoxylin and eosin; magnification: 400×.

**Figure 3:**
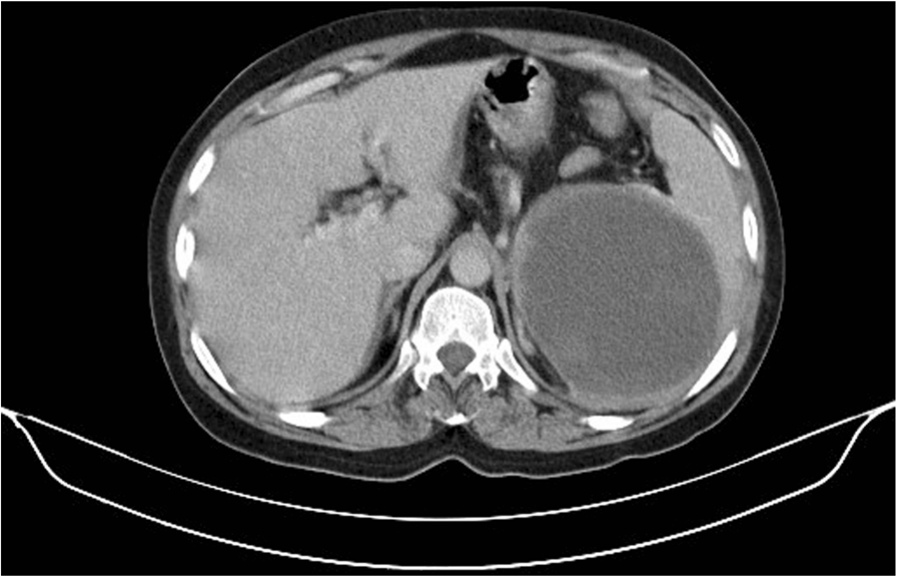
Computed tomography image showing a large hypodense splenic lesion.

We report the case of a 55-year-old North African woman, with no relevant antecedents, who presented with squamous cell uterine cervical carcinoma stage IIb according to the International Federation of Gynecology and Obstetrics (FIGO) criteria. She had no distant metastasis, and was treated with concomitant chemoradiotherapy with 46 grays on the pelvis with cisplatin (40mg/m^2^) followed by high-dose-rate intracavitary brachytherapy. The patient was cured on clinical and radiological evaluation and was on regular follow-up. She remained in a stable condition for eight months until she presented with a painful nodule in her left breast, and left upper quadrant abdominal pain. A physical examination found a hard nodule of 2cm in major axis without inflammatory signs or lymph nodes. A mammography was performed and showed a bifocal malignancy in the upper-internal quadrant of her left breast classified as Breast Imaging-Reporting and Data System category IV (BI-RADS IV). An excisional biopsy with histological study demonstrated a poorly differentiated squamous cell carcinoma with estrogen receptor negative, progesterone receptor 1% status (Figure 1, Figure 2). A pelvic examination under general anesthesia showed no suspicious lesion. The thoracoabdominal pelvic and brain computed tomography (CT) scan showed a large splenic isolated hypodense lesion sized 100mm at the largest diameter (Figure 3). There was no lymphadenopathy or other visceral involvement on the CT scan.

Based on previous history of tumor, the imaging and the histopathology findings, our patient was considered in splenic and intramammary metastatic relapse from the squamous cell carcinoma of the cervix.

She undertook a chemotherapy regimen based on paclitaxel 175mg/m^2^ and cisplatin 50mg/m^2^ every three weeks, with poor tolerance after three cycles and poor general condition, with a performance status of three according to Eastern Cooperative Oncology Group (ECOG) criteria. For that reason, we stopped chemotherapy and our patient died three months later.

## Discussion

Cervical cancer is one of the most common malignant diseases in women worldwide. The pattern of metastatic diffusion initially involves pelvic lymph nodes, followed by para-aortic nodes and then distant sites. The most frequent sites of distant recurrence are lungs, extrapelvic nodes, liver, and bones [6].

The breast is an exceptional site of metastasis from cervical carcinoma and generally occurs in widespread disease, with multiple other metastatic sites, notably lung metastases [7]. Our patient had breast and splenic metastasis, another unusual site, without other localizations.

The splenic isolated metastasis is exceptional in solid tumors, occurring approximately in 1% of the autopsy studies [8]. And squamous cervical carcinoma origin has been reported in only three cases in the literature [3-5].

The physiopathology of breast and splenic metastasis in cervical cancer is unknown; extending mechanisms through blood and lymph nodes have been suggested [9,10].

The breast metastases pose a major differential diagnosis with primary breast cancers whose management and prognosis are different. A comparison of clinical and pathological data is needed for a correct diagnosis. An immunohistochemical study if of interest, especially if the breast tumor is revealed [11]. In our patient, the history of cervical cancer was very helpful and clinically suggestive of mammary metastasis.

The management of cervical carcinoma with metastasis to the breast or spleen is not clear as it is a rare clinical entity. The majority of the reports used palliative chemotherapy.

The prognosis of breast metastasis is poor as it implies widespread tumor dissemination. Most patients die within the year following the diagnosis [12].

## Conclusions

We report the first case of the association of two uncommon metastatic sites from uterine cervix carcinoma. The clinical history of our patient helped us to establish the diagnosis, but once the metastasis is revealed, we have to be careful to distinguish the primary site of the metastasis, because treatment modality and prognosis are very different.

## Consent

Written informed consent was obtained from the patient’s next-of-kin for publication of this case report and any accompanying images. A copy of the written consent is available for review by the Editor-in-Chief of this journal.

## Abbreviations

BI-RADS: Breast Imaging-Reporting and Data System; CT: Computed tomography; ECOG: Eastern Cooperative Oncology Group; FIGO: International Federation of Gynecology and Obstetrics.

## Competing interests

The authors declare that they have no competing interests.

## Authors’ contributions

MA and SL followed the patient and wrote the manuscript. MK, AB and HA helped with the literature research. HM and HE approved the treatment and analyzed the literature data. BK performed the histological examination. All authors read and approved the final manuscript.

## Authors’ information

MA, SL, AB and ME are residents in the Medical Oncology Department at the National Institute of Oncology, Rabat, Morocco. HA is a medical oncologist at the National Institute of Oncology, Rabat, Morocco. BE is a professor of pathology at the National Institute of Oncology, Rabat, Morocco. HM and HE are professors of medical oncology at the National Institute of Oncology, Rabat, Morocco.
